# Focus on nanomedicine molecular science

**DOI:** 10.1080/14686996.2016.1181824

**Published:** 2016-05-12

**Authors:** Kazuhiko Ishihara, Nobuhiko Yui

**Affiliations:** ^a^ The University of Tokyo; ^b^ Tokyo Medical and Dental University

The development of effective and non-invasive medical treatment techniques, focusing on molecular reactions in the cells that constitute a living organism and control their biological activities, may provide new insights into intracellular molecular reactions and their parameters. These insights will help to improve medical devices, such as medical imaging equipment; they will promote computer-aided design of new drugs and reliable production of cells, including iPS cells; they may also advance research on molecular dynamics in the cellular environment, development of non-invasive diagnostic and therapeutic approaches for improving the quality of life, and growth of medical and pharmaceutical industries.

This interdisciplinary research target requires a worldwide collaboration of researchers from various fields of science and engineering, including materials science, chemistry, biology, and basic and clinical medicine. Since 2011 such collaboration was carried out within a project on “Nanomedicine Molecular Science” supported by a Grant-in-Aid for Scientific Research on Innovative Areas from the Ministry of Education, Culture, Sports, Science and Technology, Japan. This focus issue of *Science and Technology of Advanced Materials* is released on the occasion of completing the project in 2016. We would like to thank all the authors who contributed to this focus issue and hope that it will bring new ideas to their future research in the field of nanomedicine.

